# Evolution of antibody titres against Epstein–Barr virus and human herpesvirus 6A/B and expression of multiple sclerosis-associated retrovirus in the serum of pregnant multiple sclerosis patients

**DOI:** 10.1038/s41598-021-87941-1

**Published:** 2021-04-19

**Authors:** Silvia Pérez-Pérez, Juan Pablo Cuello, Marisa Martínez-Ginés, Beatriz Pardo-Rodríguez, José Manuel García-Domínguez, María Inmaculada Domínguez-Mozo, Alberto Lozano-Ros, María Ángel García-Martínez, Yolanda Higueras, Ariana Meldaña-Rivera, Haydee Goicochea-Briceño, Amalia Tejeda-Velarde, Jose Ignacio Fernández-Velasco, Silvia Medina, Rafael Arroyo, Luisa María Villar, Roberto Álvarez-Lafuente

**Affiliations:** 1 Grupo de Investigación de Factores Ambientales en Enfermedades Degenerativas, Instituto de Investigación Sanitaria del Hospital Clínico San Carlos (IdISSC) / Red Española de Esclerosis Múltiple (REEM), Madrid, Spain; 2 Servicio de Neurología, Hospital Universitario Gregorio Marañón / Red Española de Esclerosis Múltiple (REEM), Madrid, Spain; 3 Instituto de Investigación Sanitaria del Gregorio Marañón / Facultad de Psicología, Universidad Complutense de Madrid / Red Española de Esclerosis Múltiple (REEM), Madrid, Spain; 4 Instituto de Investigación Sanitaria del Gregorio Marañón / Red Española de Esclerosis Múltiple (REEM), Madrid, Spain; 5 Servicio de Inmunología, Hospital Universitario Ramón y Cajal/Instituto Ramón y Cajal de Investigación Sanitaria (IRYCIS) / Red Española de Esclerosis Múltiple (REEM), Madrid, Spain; 6 Departmento de Neurología, Hospital Universitario Quironsalud Madrid / Red Española de Esclerosis Múltiple (REEM), Madrid, Spain

**Keywords:** Predictive markers, Multiple sclerosis, Herpes virus, Retrovirus

## Abstract

Epstein–Barr virus (EBV), human herpesvirus 6A/B (HHV-6A/B) and multiple sclerosis (MS)-associated retrovirus (MSRV) have been described as possible MS triggers. We analysed antibody titres against EBV and HHV-6, and MSRV envelope (env) mRNA expression, in the serum of pregnant multiple sclerosis patients (P-MS) to study their possible link to the clinical activity of MS during pregnancy and postpartum and their possible role as relapse predictors. For that purpose, serum samples were collected from 71 pregnant women (50 pregnant MS and 21 pregnant healthy controls—P-HC) during pregnancy and postpartum. Relating to antibody titres, IgM antibody titres against HHV-6A/B were significantly higher in P-MS than in P-HC both in each pregnancy trimester and in the postpartum period. Moreover, IgM antibody titres against HHV-6A/B were higher in P-MS who suffered a relapse during the postpartum. Regarding MSRV env mRNA expression, the prevalence in the first trimester of pregnancy was significantly higher in P-MS who suffered relapses during pregnancy. Summing it up, high IgM antibody titres against HHV-6A/B and MSRV env mRNA expression during the first trimester of pregnancy could act as relapse predictors for the gestation/postpartum periods.

## Introduction

Multiple sclerosis (MS) is a chronic, autoimmune and demyelinating disease that affects the central nervous system. The most common disease course, the relapsing–remitting MS (RR-MS), is characterised by the presence of episodes of neurological dysfunction, called relapses, followed by a complete or partial recovery^1^. Although the causes of MS remain partially unknown, it is thought that environmental factors trigger MS in genetically susceptible individuals. Viruses are one of the most studied environmental risk factors for MS development. Epstein–Barr virus (EBV), human herpesvirus 6A/B (HHV-6A/B) and MS-associated retrovirus (MSRV)—a retrovirus that belongs to the human endogenous retrovirus W (HERV-W) family—have been proposed as MS risk factors^2^.

Within the clinical course of MS, pregnancy is a special period characterised by a reduction in the relapse rate. On the contrary, during postpartum, the relapse rate increases again. The causes of these facts are still unknown^3^.

Pregnancy also implies immunological changes that could alter viral immunological responses. EBV and HHV-6 titres alterations during pregnancy have been reported too^4,5^. Moreover, the envelope protein (env) of a member belonging to the HERV-W family, the syncytin-1, is implied in syncytiotrophoblast formation during placentation^6^. Syncytin-1 and MSRV env (the envelope protein of the complete viral particle) show 94% homology, sharing several biological properties^7^.

In this work, we aimed to analyse EBV and HHV-6A/B titres and MSRV env mRNA presence in serum samples from MS patients and healthy controls during pregnancy and postpartum and to relate them with clinical activity of the disease measured by relapse rate during gestation and postpartum, and also their potential role as relapse predictor.

## Results

**Figure 1:**
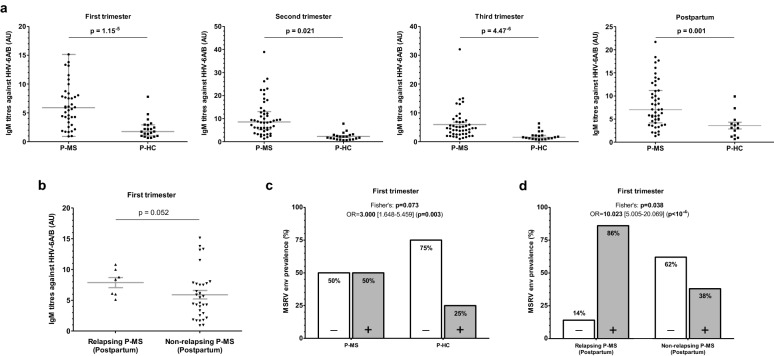
Antibodies titres and MSRV env expression. (**a**) IgM antibody titres against HHV-6A/B were significantly higher in P-MS compared to P-HC during both pregnancy and postpartum. (**b**) Considering P-MS, IgM antibody titres against HHV-6A/B were higher in those who suffered relapses during postpartum compared to those who did not. (**c**) MSRV env expression prevalence was higher in P-MS than in P-HC during the first trimester of pregnancy. (**d**) Moreover, MSRV env expression prevalence was significantly higher in P-MS who suffered relapses during pregnancy compared to those who did not. (A, B: U-Mann Whitney’s test; C, D: Fisher’s exact tests, OR) AU: arbitrary units. Graphs made with Prism 5.00 (GraphPad).

IgM antibody titres against HHV-6A/B were significantly higher in P-MS than in P-HC both in each pregnancy trimester and in the postpartum period. Moreover, in the first trimester of pregnancy, anti-HHV-6A/B IgM antibody titres were also higher in those P-MS who relapsed during the postpartum compared to those who did not (Fig. 1a,b).

Regarding MSRV env mRNA expression (Fig. 1c,d), the prevalence in the first trimester of pregnancy was higher in P-MS than in P-HC—showing a trend towards significance. Furthermore, also during the first trimester of pregnancy, the positivity percentage was significantly higher in P-MS who relapsed during the gestation period compared to those who did not suffer relapses.

Furthermore, we performed the same statistical analyses comparing those P-MS that suffered severe and non-severe outbreaks, as well as comparing those P-MS with disease progression (increase in disability), as a consequence of the relapses suffered, and those without it. We did not find statistically significant differences in any of the cases.

As regards the results of IgG antibodies against EBV (both anti-VCA and anti-EBNA-1) and IgG antibodies against HHV-6A/B, there were no statistically significant differences in any of the test performed (Supplementary Table S1 shows every p-value obtained).

## Discussion

MS is the leading cause of non-traumatic disability in young adults worldwide^8^, which means that many of the female patients suffering the disease were, are, or will be considering getting pregnant. Moreover, pregnancy is a special period within the clinical course of MS, in which patients experience a decrease in the clinical activity of the disease^3^. Therefore, it is essential to understand the mechanisms underlying this special period and to know how to modulate treatments during gestation, both to protect the mother and the unborn child, since some MS treatments have been reported to involve teratogenic or severe side effects for them.

In this line, one of the main issues to investigate is the possibility of having a relapse biomarker, which allows physicians to better treat the patient in case of being at risk of relapse or, on the contrary, to allow the patient to stop her treatment during pregnancy and/or postpartum without—or at least with very little—risk.

In this study, we have found that IgM antibodies against HHV-6A/B could act as a biomarker of relapse suffering during postpartum since titres during the first trimester are significantly higher in P-MS who experienced a relapse compared to those who did not. Other studies support this result, in which there was an increment in IgM antibody titres against HHV-6A/B a few days before the relapse^9^. Although in this study we have analysed antibody titres against HHV-6A/B together, using ELISA commercial kits available at the time we performed the aforementioned experiments, a recently published paper has analysed the titres against HHV-6A and HHV-6B separately, using a novel multiplex serological assay. Therefore, it would be of great interest to determine in future studies whether HHV-6A or HHV-6B are involved in the risk of relapse during postpartum^10^.

Moreover, we have also found that MSRV env mRNA expression in the first trimester of pregnancy is significantly more prevalent in those women with relapses during gestation. This finding is in line with many others published before where the expression of MSRV is related to a higher relapse rate^7,11,12^. Therefore, these results seem to show that MSRV could be playing a role in relapse development.

Despite the results obtained with the levels of IgM anti-HHV-6A/B, we did not find that level of significance with any other serology. IgG antibodies do not reflect an active infection/viral reactivation, and this could explain why IgM anti-HHV-6A/B could be a better biomarker for relapse risk. As regards anti-EBV antibodies, while there is considerable evidence of the involvement of EBV in MS neurodegeneration, its link to relapses is unclear^13–15^.

Finally, although we have studied several variables and performed the corresponding tests, we have not applied corrections for multiple comparisons due to the exploratory nature of this study.

Taken together, these results seem to indicate that both elevated IgM antibody titres against HHV-6A/B and the presence of MSRV env mRNA in serum during the first trimester of pregnancy are potential predictors of relapse risk during the postpartum or the gestation periods, respectively. The possibility of finding a risk relapse biomarker for pregnant women is extremely needed because of its important clinical and therapeutic implications. However, these results must be taken with caution and more comprehensive and larger studies are needed to validate them.

## Materials and methods

### Patients and samples

We performed a prospective study including a total of 71 pregnant women (Table 1). This cohort comprised 50 pregnant MS patients (P-MS) diagnosed at Hospital General Universitario Gregorio Marañón (Madrid, Spain), according to McDonald criteria^16^, and 21 pregnant healthy controls (P-HC). The study was approved by the Ethics Committee (Comité Ético de Investigación Clínica del Hospital General Universitario Gregorio Marañón—CPMP/ICH/135/95) and it was conducted following the Declaration of Helsinki. All patients signed informed consents.

**Table 1:** Demographical characteristics of the patients included in the study.

	P-MS	P-HC
n	50	21
Age at conception (years old, median (P25–P75))	33.0 (31.0–36.0)	34.0 (29.0–36.0)
Disease duration (years, median (P25–P75))	9.0 (5.0–13.0)	–
EDSS at conception (median (P25–P75))	1.0 (0.0–1.0)	–
Relapses during pregnancy (%)	18.0	–
Relapses during postpartum (%)	22.0	–
**Treatment before pregnancy (%)**		
First line therapy (interferon beta, glatiramer acetate)	50.0	–
Second line therapy (natalizumab, fingolimod)	12.0	–
Without treatment	38.0	–

*P-MS* pregnant MS patients, *P-HC* pregnant healthy controls, *EDSS* expanded disability status scale.

Serum samples were collected in dry tubes from each patient in each trimester of pregnancy (weeks 8–12, 13–28, 29–37) and in the postpartum period (first trimester after birth). After collection, they were centrifuged (900×*g*, 15′, room temperature) and stored at − 80 °C.

### Enzyme-linked immunosorbent assay (ELISA)

We used Captia (Trinity Biotech, Wicklow, Ireland) tests for the detection of IgG against the viral capsid antigen (VCA) and the nuclear antigen 1 (EBNA-1) of EBV, and Vircell (Granada, Spain) tests for the detection of IgG and IgM against HHV-6A/B, following the manufacturer’s instructions, including the use of negative controls and standards.

### MSRVenv expression

Viral RNA extraction was carried out in pellets obtained after serum samples ultracentrifugation (352,270, 62×*g*, 20′, 8 °C) using the QIAamp Viral RNA Kit (Qiagen, Valencia, CA, USA). Viral RNA was treated using the TURBO DNA-free Kit (Invitrogen, Carlsbad, CA, USA) and cDNA was obtained using the Transcriptor First Strand cDNA Synthesis Kit (Roche Diagnostics, S. L., Barcelona, España). For MSRV env expression, qPCR assays were performed using TaqMan PreAmp Master Mix (Applied Biosystems, Foster City, CA, USA) and primers and probes previously described^17^, in a Rotor Gene 3000 (Corbett Research, Mortlake, Australia). In each step, negative controls were carried out.

### Statistics

We analysed differences in viral titres and MSRV env qualitative expression between P-MS and P-HC, as well as between P-MS with and without relapses during pregnancy and/or postpartum, using Mann–Whitney U and Fisher’s exact tests (for quantitative and categorical variables, respectively)—the population was non-parametric according to Kolmogorov–Smirnov test. We also calculated the OR (odds ratio) for categorical variables. Analyses were performed using SPSS 15.0 software (SPSS Inc., Chicago, IL, USA) and statistically significant differences were considered when p < 0.05.

## Supplementary Information

Supplementary Information.

## Data Availability

The data that support the findings of this study are available on request from the corresponding author. The data are not publicly available due to privacy or ethical restrictions.
